# *Desmozoon lepeophtherii *n. gen., n. sp., (Microsporidia: Enterocytozoonidae) infecting the salmon louse *Lepeophtheirus salmonis *(Copepoda: Caligidae)

**DOI:** 10.1186/1756-3305-2-58

**Published:** 2009-11-27

**Authors:** Mark A Freeman, Christina Sommerville

**Affiliations:** 1 Institute of Aquaculture, University of Stirling, Stirling, FK9 4LA, UK; 2 Institute of Earth and Ocean Sciences, University of Malaya, Kuala Lumpur, 50603, Malaysia

## Abstract

**Background:**

A microsporidian was previously reported to infect the crustacean parasite, *Lepeophtheirus salmonis *(Krøyer, 1837) (Copepoda, Caligidae), on farmed Atlantic salmon (*Salmo salar *L.) in Scotland. The microsporidian was shown to be a novel species with a molecular phylogenetic relationship to *Nucleospora *(Enterocytozoonidae), but the original report did not assign it to a genus or species. Further studies examined the development of the microsporidian in *L. salmonis *using electron microscopy and re-evaluated the molecular findings using new sequence data available for the group. Here we report a full description for the microsporidian and assign it to a new genus and species.

**Results:**

The microsporidian infects subcuticular cells that lie on the innermost region of the epidermal tissue layer beneath the cuticle and along the internal haemocoelic divisions. The mature spores are sub-spherical with a single nucleus and an isofilar polar filament with 5-8 turns in a double coil. The entire development is in direct contact with the host cell cytoplasm and is polysporous. During early merogony, a diplokaryotic nuclear arrangement exists which is absent throughout the rest of the developmental cycle. Large merogonial plasmodia form which divide to form single uninucleate sporonts. Sporogonial plasmodia were not observed; instead, binucleate sporonts divide to form two sporoblasts. Prior to final division, there is a precocious development of the polar filament extrusion apparatus which is associated with large electron lucent inclusions (ELIs). Analyses of DNA sequences reveal that the microsporidian is robustly supported in a clade with other members of the Enterocytozoonidae and confirms a close phylogenetic relationship with *Nucleospora*.

**Conclusion:**

The ultrastructural findings of the precocious development of the polar filament and the presence of ELIs are consistent with those of the Enterocytozoonidae. However, the confirmed presence of an early diplokaryotic stage and a merogonial plasmodium that divides to yield uninucleate sporonts instead of transforming into a sporogonial syncitium, are features not currently associated with the family. Yet, analyses of DNA sequence data clearly place the microsporidian within the Enterocytozoonidae. Therefore, due to the novelty of the copepod host, the ultrastructural findings and the robust nature of the phylogenetic analyses, a new genus should be created within the Enterocytozoonide; *Desmozoon lepeophtherii *n. gen. n. sp. is proposed.

## Background

Microsporidia infect a wide range of invertebrate and vertebrate taxa, and are common parasites of both fish and arthropods. Reports of microsporidian infections in marine and freshwater crustacea are also numerous and include various microsporidian genera infecting marine crabs [1-4], brine shrimp [5], cladocerans [6,7], ostracods [8], marine shrimps [9-14], amphipods [15], freshwater crayfish [16], marine lobster [17] and copepods [18-22].

Although nine genera and approximately 50 species of microsporidia have been described from copepods [18,20,22], none have been described from free-living marine copepods. However, there are two reports of microsporidia as hyperparasites of parasitic marine copepods. An unidentified microsporidian, phylogenetically related to *Nucleospora *spp. (Enterocytozoonidae), has been reported to infect the caligid copepod *Lepeophtheirus salmonis *parasitic on farmed Atlantic salmon, *Salmo salar *[21,23], and *Unikaryon mytilicolae *(Durfort, Vallmitjana & Vivares, 1980) has been found infecting the cyclopoid copepod *Mytilicola intestinalis *(Steuer, 1902) on marine mussels [24]. A single report also exists for the hyperparasitic microsporidian, *Microsporidium lamproglenae*, infecting the freshwater copepod *Lamproglena pulchella *(von Nordmann, 1832) parasitic on the cyprinid fish, *Leuciscus leuciscus *L. in Europe [25].

The majority of microsporidians described from free-living freshwater copepods have complex life cycles utilising copepods as intermediate hosts and mosquito larvae as definitive hosts [26-28]. The life cycles of the microsporidians infecting parasitic marine copepods are not fully known. However, the hyperparasitic microsporidian infecting *L. salmonis *was shown experimentally not to transmit directly from copepod to copepod. Furthermore, the microsporidian DNA was isolated from internal tissues from the fish host, suggesting that the Atlantic salmon may be involved in the life cycle [23].

The microsporidian hyperparasite from *L. salmonis *was placed in the Enterocytozoonidae by Freeman *et al*. [21] based on the results of robust phylogenetic analyses, but they did not assign it to a genus. At that time, only two genera existed in the Enterocytozoonidae. *Enterocytozoon*, containing a single species, *Enterocytozoon bieneusi *(Desportes, 1985), an intestinal parasite of humans and other mammals [29,30] and now known to be comprised of numerous genotypes infecting a wide range of mammals and birds [31]. The second genus, *Nucleospora*, is comprised of intranuclear microsporidians infecting fish [32-34]. The microsporidian from *L. salmonis *could not be placed in either the *Enterocytozoon *or the *Nucleospora *as the host species was too dissimilar and it was neither an intranuclear microsporidian nor one infecting intestinal enterocytes. In addition, the small round to sub-spherical spores of the microsporidian from *L. salmonis*, measuring 2.34 × 1.83 μm (± 0.01 μm), developing without sporophorous vesicles and forming large xenomas (up to 300 μm in diameter) in the sea lice hosts were sufficiently unique features to enable it to be distinguished from all previously described microsporidia from copepods in freshwater as well as microsporidia infecting other marine crustacea [21].

Since Freeman *et al*. [21] assigned the microsporidian from *L. salmonis *to the family Enterocytozoonidae, a new genus, *Enterospora*, has been created in the family for intranuclear microsporidia infecting the hepatopancreatocytes of marine decapods [4] and a new species *Enterocytozoon hepatopenaei*, only the second species in the genus, has been described from the cytoplasm of the tubule epithelial cells of the hepatopancreas in the black tiger shrimp *Penaeus monodon *(Fabricius, 1798) from Thailand [14]. Thus, the Enterocytozoonidae currently contains three genera: *Enterocytozoon *spp. that infect the cell cytoplasm of mammalian and bird enterocytes and tubule epithelial cells of the hepatopancreas in the black tiger shrimp, *Nucleospora *spp. that infect the nucleus of various fish cells and *Enterospora *spp. that infect the nucleus of hepatopancreatocytes in marine decapods.

In the present study we examine the ultrastructure of the microsporidian infecting *L. salmonis *from farmed Atlantic salmon and provide an updated molecular phylogenetic analysis. Using the combined data we describe *Desmozoon lepeophtherii *n. gen. n. sp., and place it in the Enterocytozoonidae as a fourth genus of the family.

## Methods

Salmon lice, *Lepeophtheirus salmonis*, were collected from salmon farms on the West Coast of Scotland. Adult lice were carefully removed using forceps from recently culled fish during routine harvests. Specimens were transferred from the farm site to the laboratory in bags of seawater at ambient temperature and salinity. Once in the laboratory, specimens were maintained in seawater collected from the farm site, aerated at 10°C in 10 L aquaria. Daily removal of senescent and dead lice with 80% water change allowed the remaining lice to survive without their hosts for several days.

Lice were screened for the presence of microsporidian infection using a dissecting microscope. Lice with inclusions under the carapace were dissected and fresh squash preparation observed to confirm the presence of microsporidian spores using a compound microscope. Confirmed infected lice were dissected further as required and fixed in 2.5% glutaraldehyde for 2 hours, washed in cacodylate rinse buffer (0.1 M, pH 7.2) overnight. Samples were then post-fixed in 1% osmium tetroxide (in 1% borax solution) for one hour, before dehydration through a graded acetone series. After dehydration to 100% acetone, the samples were transferred to a 1:1 mix of Spurr resin [35] and acetone for 1 hr on a rotator followed by a further 2 hr in a mix of 3:1 Spurr: acetone. Finally, the specimens were rotated for 24 hr in 100% Spurr resin before being embedded in Beem capsules and polymerised at 60°C for 48 hrs.

All sections were cut using a glass knife on a Reichert Ultracut E ultramicrotome. Semi-thin sections of 1 μm were first cut and visualised by staining with 1% alcian blue for 5 min and examined under light microscopy. Ultrathin sections (80 nm) were mounted on 200 mesh Formvar coated copper grids and stained with uranyl acetate and lead citrate. Ultrathin sections were examined with a Philips 301 TEM operating at 80 kV. Photographs were taken with a flat plate camera using black and white Kodak 4489 EM film.

Whole infected lice were frozen in liquid nitrogen and fractured whilst under nitrogen, by applying pressure to the carapace with a scalpel. Fractured lice were then fixed at 4°C for one hour in 1% gluteraldehyde in 0.1 M cacodylate buffer. This initial fixation was followed by a 2-3 day immersion in 3% gluteraldehyde in 0.1 M cacodylate buffer at 4°C, followed by an over night rinse in 0.1 M cacodylate buffer. Samples were then post-fixed in 1% osmium tetroxide (in 1% borax solution) for 2 hrs, before dehydration through a graded ethanol series. Once in 100% ethanol, samples were transferred to a 50:50 mix of ethanol and hexamethyldisilazane (HMDS) for 30 min before being transfered to 100% HMDS for a further 30 min. Samples were air-dried at room temperature over night, mounted on aluminium stubs and coated with gold at 40 mA for 90s using an Edwards S150B Sputter Coater. Samples were examined using a Philips 500 scanning electron microscope operating at 15 kV. Photographs were taken using an integral camera with Ilford FP4-125 film.

Representative small subunit ribosomal DNA (SSU rDNA) sequences from the Enterocytozoonidae were used in order to view the relatedness of genera and species within the family. Sequences used are available in GenBank under the following accession numbers: *Desmozoon lepeophtherii *AJ431366; *Enterocytozoon bieneusi *L07123; *Enterocytozoon bieneusi *DQ793212; *Enterocytozoon hepatopenaei *FJ496356; *Nucleospora salmonis *U10883; *Nucleospora salmonis *AF186003; *Nucleospora salmonis *AF185989; *Nucleospora salmonis *AF186006; *Nucleospora *sp. AF186007; *Microsporidium *sp. AF394528; *Microsporidium *sp. FJ794872. Sequences were aligned using ClustalX [36] and percentage divergence matrices constructed using the Neighbour-joining method [37]. The same alignment files were used to construct phylogenetic trees using heuristic searches and maximum parsimony in PAUP*4.0 beta10 [38].

In accordance with section 8.6 of the ICZN's International Code of Zoological Nomenclature, copies of this article are deposited at the following five publicly accessible libraries: Natural History Museum, London, UK; American Museum of Natural History, New York, USA; Museum National d'Histoire Naturelle, Paris, France; Russian Academy of Sciences, Moscow, Russia; Academia Sinica, Taipei, Taiwan.

## Results

**Figure 1:**
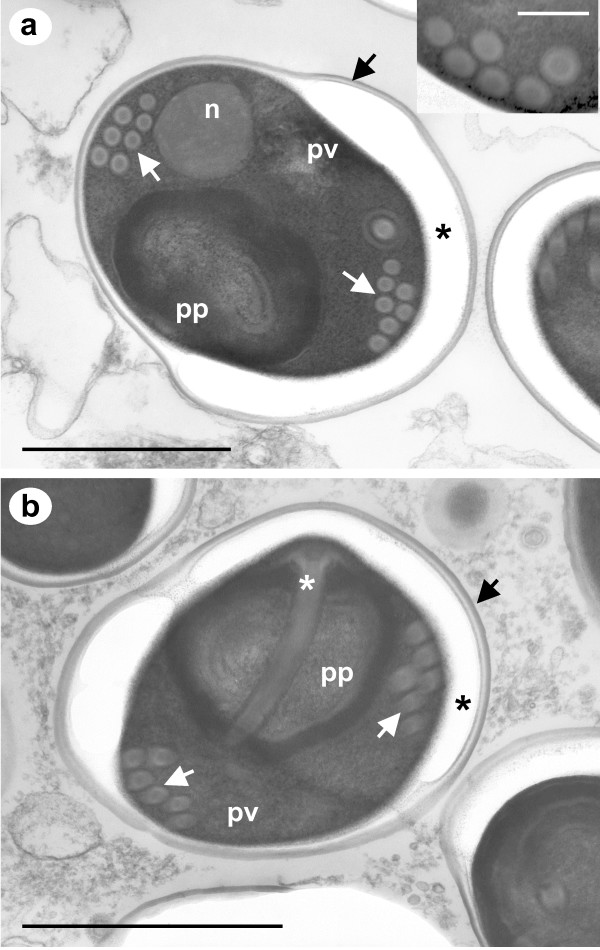
**a-b - TEM of mature spores of *Desmozoon lepeophtherii *n. gen., n. sp**. Sagittal section of spores detailing seven coils of an isofilar polar filament (white arrows) in a double coil arrangement. The polar filament has an electron dense core and is composed of concentric layers of varying electron densities (inset a). A thick electron lucent endospore wall (black asterisks) is surrounded by a thinner electron dense exospore layer (black arrows). The single nucleus (n), posterior vacuole (pv) and polaroplast (pp) are all prominent features of the mature spore. The polaroplast is located at the anterior end of the spore and accommodates the manubroid part of the polar filament (white asterisk) and the attachment disc. Scale bars 1 μm (200 nm inset a).

Fresh wet-mount preparations from dissected *L. salmonis *with inclusions under the carapace revealed the presence of numerous microsporidian-like spores. A definitive identification of microsporidian spores was confirmed with TEM of the infected lice. Mature spores are sub-spherical (round to ovoid), with a single nucleus and a thickened electron lucent endospore wall measuring between 150-250 nm thick surrounded by a thinner electron dense exospore measuring between 35-40 nm thick (Fig. 1a-b). The polar filament has between 5 and 8 turns, normally in a double coil, and is of the isofilar type, being of a similar diameter (65-85 nm) along its entire length. The mature spore has typical features of a microsporidian spore. The polar filament is attached at the anterior end of the spore via the anchoring disc, which is surrounded by the laminar structure of the polaroplast; the posterior vacuole is located at the opposite end of the spore.

**Figure 2:**
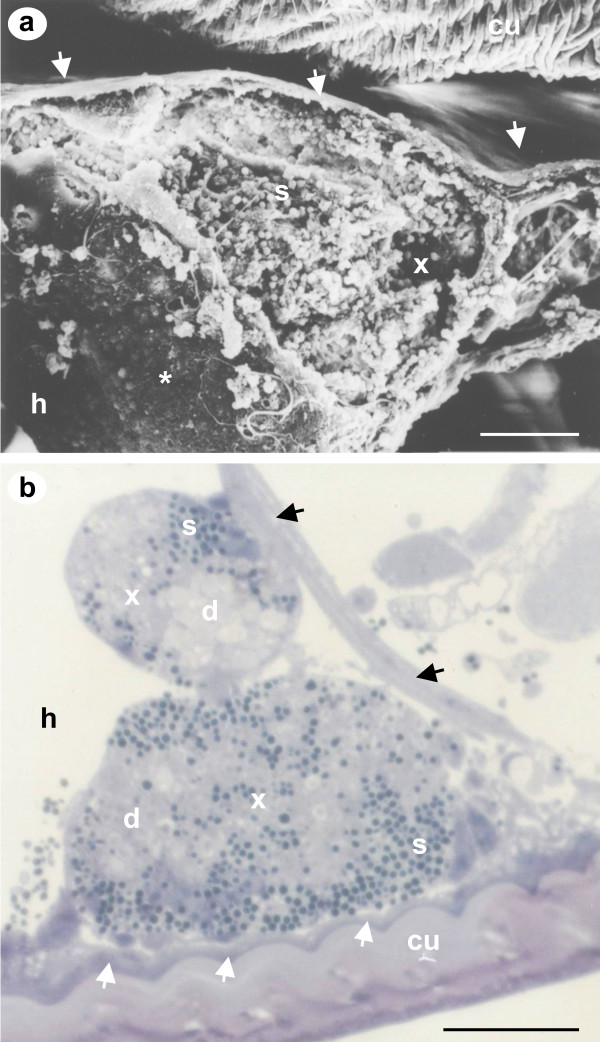
**SEM of freeze-fractured *L. salmonis *and transverse abdominal semi-thin histological section**. a) SEM of freeze-fractured louse showing a large xenoma (x) developing beneath the cuticle (cu). The epidermal tissue layer remains uninfected directly beneath the cuticle (white arrows) but has a different more coarse appearance (white asterisk) bordering the haemocoel (h). The xenoma has been fractured open revealing that it is packed with microsporidian spores (s). b) Transverse abdominal semi-thin section from an infected louse. Developing xenomas (x) contain both mature spores (s) and developing stages (d) and can originate from beneath the cuticle (cu) or from the haemocoelic divisions that separate the haemal sinuses (black arrows). The epidermal tissue layer beneath the cuticle remains intact (white arrows). Scale bars a 25 μm, b 50 μm.

**Figure 3:**
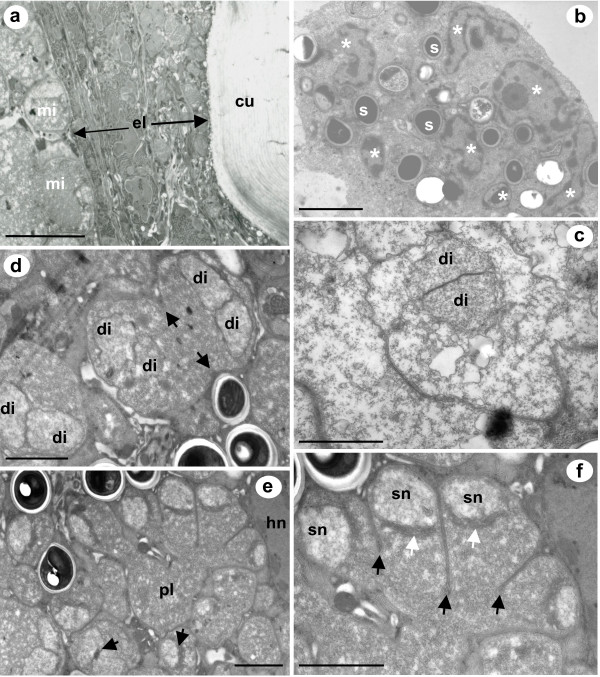
**TEM of microsporidian infection beneath the cuticle, xenoma structure and merogonial replication of *Desmozoon lepeophtherii *n. gen., n. sp**. a) The microsporidian infection (mi) is found beneath the cuticle (cu) and originates from the innermost portion of the epidermal tissue layer (el). b) A small xenoma contains mature spores (s) and reveals that the host nucleus (white asterisks) is not infected by the microsporidian but is grossly hypertrophic and has numerous branches and folds. c) An early meront stage with a single nucleus in diplokaryotic formation (di). d) A divisional meront showing cytoplasmic constrictions (black arrows), that contains two nuclei in diplokaryotic arrangement (di). e) A large divisional merogonial plasmodium (pl) situated next to a host cell nucleus (hn), black arrows indicate nuclear activity associated with nuclear dissociation of the diplokaryon in neighbouring meronts. f) An enlarged view of the same plasmodium, with single unpaired nuclei (sn), undergoing division via plasmotomy (black arrows). White arrows indicate electron-dense laminate bodies lying on the nuclear membrane indicating recent nuclear activity due to nuclear dissociation of the diplokaryotic arrangement. Scale bars a-b 5 μm, c-f 2 μm.

SEM of freeze-fractured *L. salmonis *revealed large xenomas developing under the cuticle (Fig. 2a). The cellular surface of the epidermal layer adjacent to the cuticle is smooth and uninfected, whilst the inner most sub-cuticular epidermal region adjoining the haemocoel that contains the xenoma, has a coarse and pitted appearance (Fig. 2a). A semi-thin abdominal histological section shows xenomas forming from the innermost surface of the epidermal layer beneath the cuticle and also from the structures separating the haemal sinuses in the haemocoel (Fig. 2b). The cells in this region consist mainly of desmocytes, fibrocytes and tissue-dwelling haemocytes. The exact host cell type was undistunguishable owing to the changes effected during xenoma formation. However, the microsporidian appears to infect cells that are associated with a glycocalyx-like border on the innermost part of the epidermal layer bordering the haemocoel and along haemocoelic divisions between sinuses. TEM confirms that infection was not observed throughout the epidermal layer and is restricted to the innermost surface (Fig. 3a).

Occasional host cell remnants could be seen in large xenomas but smaller xenomas contained multiple nuclei, nuclear fragments or multi-lobed nuclei (Fig. 3b); the microsporidian was never observed to have a close association with the host cell nucleus or seen as intranuclear at any stage of development. The microsporidian remained in direct contact with the host cell cytoplasm throughout the developmental cycle and did not form a complete interfacial envelope of either parasite-derived or host-derived origin that contained a typical number of spores.

Figure 3c is an early meront and is the earliest microsporidian stage that was observed. The nucleus is in a diplokaryotic arrangement and the cytoplasm is electron lucent lacking other cellular organelles. The diplokaryotic nuclei of the early meronts divide to produce two diplokaryotic nuclei which locate at opposing cellular borders (Fig 3d). Cytokinesis follows this nuclear division and the cell divides by binary fission, producing two new meronts each with a diplokaryon (Fig 3d); during this division the cytoplasm has a much more granular appearance. The diplokaryotic nuclear arrangement was not observed after this point in the developmental phase and nuclear dissociation is assumed to have occurred. Nuclear dissociation is followed by multiple subsequent nuclear divisions, which are not directly linked to cytokinetic events, resulting in the formation of a large, paucinucleate, rounded, merogonial plasmodium, which ultimately divides by plasmotomy (Fig 3e). During division, the plasmodium contains very granular cytoplasm with no obvious organisation of organelles and the nuclei have electron-dense laminate bodies lying on the nuclear membrane, indicative of recent nuclear activity from either nuclear dissociation or recent division (Fig 3f).

**Figure 4:**
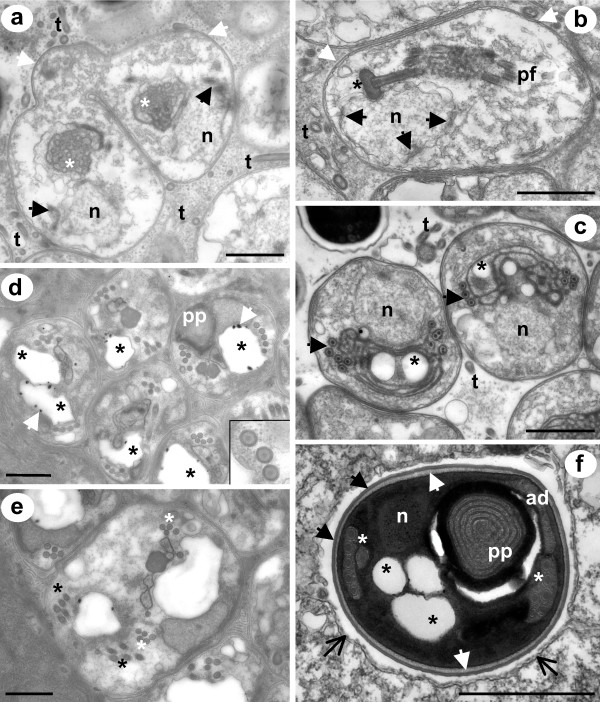
**TEM of sporogonial stages of *Desmozoon lepeophtherii *n. gen., n. sp**. a) An early divisional sporont with tubules in the host cell cytoplasm has a modest thickening of the plasma membrane (white arrows) and precocious development of the pf (black arrows) associated with the nucleus and cytoplasmic cisternae (white asterisks) that start to form the pf bundles. b) Sporont with a thickening plasma membrane (white arrows), a diffuse nucleus showing signs of recent activity (black arrows), the developing ad (black asterisks) is associated with a nuclear invagination, from which the developing pf extends. Tubules are present in the host cell cytoplasm. c) Immature sporoblasts have a more defined nucleus and show signs of pf organisation in to bundles (black arrows), which are arranged in close proximity to large ELIs (black asterisks), tubules are still present in the host cell cytoplasm. d) Maturing sporoblasts have features of mature spores such as a pp and a more mature pf. ELIs (black asterisks) have dark granules associated with the membranes (white arrows). e) Some sporoblasts at an equivalent developmental stage to (d) have two sets of pf apparatus (white/black asterisks) both with associated ELIs. f) An early spore with a fully formed exospore layer (black arrows) and a developing endospore layer (white arrows). This late stage shows a typical internal arrangement seen in mature spores; ad and pp are located at the anterior of the spore, the single nucleus and rows of pf (white asterisks) are medially positioned, ELIs (black asterisks) are posteriorly located and will form part of the posterior vacuole. A secretion from the exospore forms a fragile interfacial envelope which creates a void surrounding the spore (open black arrows). All scale bars 1 μm. ad (anchoring disc); n (nucleus); pf (polar filament); pp (polaroplast); t (tubules).

The resulting uninuculeate stages represent the production of sporonts and the onset of sporogony. Sporonts have a higher degree of cellular organisation than meronts. Early sporonts have a precocious development of the polar filament which is associated with the nucleus and a complex of cytoplasmic cisternae that coalesce to form the polar filament bundles; there is a modest thickening of the plasmalemma prior to division (Fig 4a). During sporogony the polar filament and its attachment apparatus, the anchoring disc, continue to develop and the plasma membrane continues to thicken (Fig 4b). The early formation of the anchoring disc is often seen to take place in association with a nuclear invagination (Fig 4b). During these preliminary stages of sporogony, numerous tubules are present in the host cell cytoplasm (Figs 4a-4c). The coils of the polar filament continue to mature with an electron dense core forming and membrane bound electron lucent inclusions appear in the posterior part of the spore between the developing bundles of polar filament (Fig 4c). These stages are considered to be sporoblasts that continue to develop into spores without further division. As the sporoblasts mature, the polar filament continues to become more electron-dense and show concentric patterns of electron density (inset Fig 4d). The ELIs become more prominent and are often associated with large electron dense particles located at the membrane (Fig 4d). Occasionally, sporoblast-like forms at a similar developmental stage retain two sets of polar filaments (Fig 4e). Immature spores start to reveal typical microsporidian spore-like features (Fig 4f). The exospore is fully formed before the endospore develops and the ELIs are now associated with the developing posterior vacuole. At this stage a fragile interfacial envelope can sometimes be seen surrounding the immature spore (Fig 4f).

**Figure 5:**
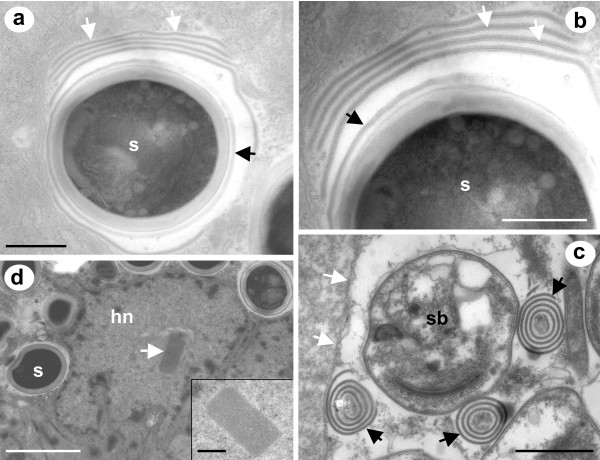
**Other ultrastructural observations associated with *Desmozoon lepeophtherii *n. gen., n. sp**. a and b) Secretions from mature spores (s) are sometimes observed that form multiple and regular layers which have a fine electron dense core, and surround the spore (white arrows). The secretions originate from the exospore surface and, when first produced, do not have an electron dense core (black arrows). c) At the border of a xenoma (white arrows) concentric whorls of the secretions have formed adjacent to a sporoblast (sb), the individual layers appear more compact but still maintain an electron dense core (black arrows). d and inset) Host cell nuclei of microsporidian-infected cells were frequently observed with intranuclear inclusions, which were of a crystalline appearance with a regular pattern. Scale bars a, b and d (inset) 500 nm, c 1 μm, d 2 μm.

Membranous-like secretions were sometimes observed in the host cell cytoplasm during sporogony (Fig 5a-c). These secretions between 30-40 nm in thickness contained a central core of electron dense particles and appeared to develop at the external surface of maturing spores. The membranous-like secretions were occasionally seen free in the host cell cytoplasm as enclosed concentric membranous whorls (Fig 5c).

Host cell nuclei of microsporidian-infected cells were sometimes observed with intranuclear inclusions, which were of a crystalline appearance having a regular pattern (Figs 5d).

From the percentage divergence matrices (Table 1), *D. lepeophtherii *is most similar to members of the *Nucleospora*, ranging from 10.0-11.8% divergence over approximately 850 bases of SSU sequence data; *Enterocytozoon *spp. are more distant at 18.3-20.2% divergence. Percentage divergence within the *Nucleospora *was as high as 14.1% between species infecting non-salmonids (English sole and Atlantic halibut) and as low as 0.3% between salmonid species. Percentage divergence between the two species of *Enterocytozoon *ranged from 13.7-20.2% depending on the genotype of *E. bieneusi*, and 1.5% between the two genotypes examined. *Desmozoon lepeophtherii *is most similar to the *Nucleospora *sp. infecting English sole with 10.0% divergence over 847 bases of the SSU examined

**Table 1:** Percentage divergence of SSU rDNA sequences for members of the Enterocytozoonidae and related taxa.

	**1**.	**2**.	**3**.	**4**.	**5**.	**6**.	**7**.	**8**.	**9**.	**10**.	**11**.
**1**. *Desmozoon lepeophtherii*		0.202	0.183	0.188	0.118	0.112	0.100	0.114	0.116	0.251	0.256

**2**. *Enterocytozoon bieneusi *(human)	827		0.015	0.202	0.167	0.169	0.195	0.174	0.184	0.223	0.261

**3**. *Enterocytozoon bieneusi *(bird)	458	845		0.137	0.147	0.147	0.179	0.151	0.143	0.203	0.239

**4**. *Enterocytozoon hepatopenaei*	690	819	607		0.171	0.171	0.203	0.174	0.164	0.233	0.264

**5**. *Nucleospora salmonis *(CS)	851	1247	855	836		0.001	0.121	0.003	0.005	0.212	0.249

**6**. *Nucleospora salmonis *(RT)	845	1228	855	836	1250		0.120	0.003	0.005	0.214	0.249

**7**. *Nucleospora *sp. (ES)	847	1228	853	839	1247	1246		0.123	0.141	0.226	0.254

**8**. *Nucleospora salmonis *(AS)	814	1199	854	835	1220	1219	1216		0.009	0.221	0.253

**9**. *Nucleospora salmonis *(AH)	671	756	561	774	776	776	775	775		0.247	0.256

**10**. *Microsporidium *sp. (DP)	872	1246	854	834	1270	1245	1244	1215	772		0.030

**11**. *Microsporidium *sp. clone B	708	1059	826	834	1076	1076	1074	1075	773	1079	

Distances (percentage divergence/100) above the diagonal and number of bases of SSU rDNA analysed below the diagonal

AH = Atlantic halibut; AS = Atlantic salmon; CS = chinook salmon; DP = *Daphnia*; ES = English sole; RT = rainbow trout

**Figure 6:**
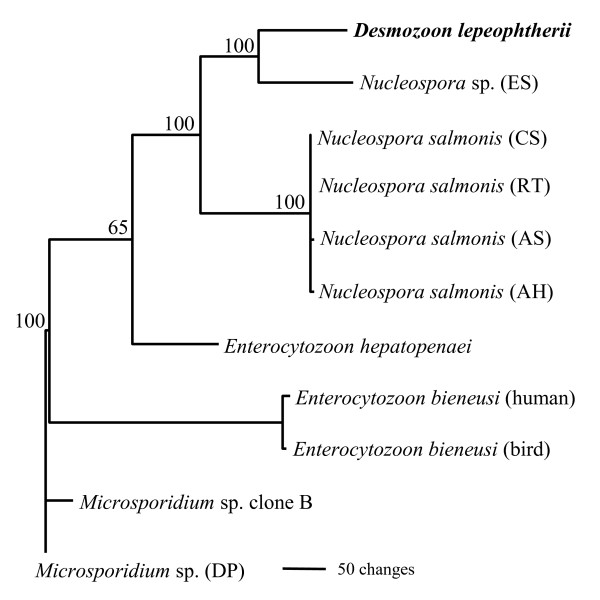
**Maximum parsimony generated phylogenetic tree constructed using SSU rDNA sequence alignments from representative taxa of the Enterocytozoonidae**. The tree utilises 328 parsimony informative characters and is rooted to the related *Microsporidium *sp. infecting *Daphnia*. The scale represents number of base changes and the numbers at the nodes indicate bootstrap support values from 1000 resamplings. AH = Atlantic halibut; AS = Atlantic salmon; CS = chinook salmon; DP = Daphnia; ES = English sole; RT = rainbow trout.

The phylogenetic tree (Fig 6) confirms that the results obtained with percentage divergence matrices were also observed using maximum parsimony analyses. *Desmozoon lepeophtherii *forms a well-supported clade with the *Nucleospora *sp. infecting English sole which forms as a sister group to the *N. salmonis *clade. The bootstrap support for these clades is 100%. *Enterocytozoon *spp. are more basal in the tree and *E. hepatopenaei *does not group with the *E. bieneusi *genotypes, but forms a solitary branch between *E. bieneusi *and the *Nucleospora *clade containing *Desmozoon*. The outgroup *Microsporidium *sp. (DP), is a microsporidian that infects the intestinal wall of *Daphnia*, is related to other *Daphnia*-infecting species such as *Microsporidium *sp. clone B [7], and has been shown to be immediately basal to the family Enterocytozoonidae in independent phylogenetic analyses [6,21].

## Discussion

### Tissue location of Desmozoon lepeophtherii in L. salmonis

The epidermal layer in copepods is described as a flattened cellular layer containing a great number of membrane-bound vesicles and mitochondria, with a very complicated spatial arrangement [39]. This complex arrangement of cells was confirmed to be similar in *L. salmonis *in the present study (Fig 3a). The basal portion of the epidermal layer rests on a glycocalyx [39], which is also referred to as the basal lamina and functions as a basement membrane separating the epidermal layer from the underlying tissues and haemocoel [40]. The glycocalyx contains epidermal cells such as modified epithelial cells with tonofilaments, desmocytes or fibrocytes and tissue dwelling haemocytes [39]. Microsporidian xenomas develop from this location, but due to the extreme hypertrophy and transformation of infected host cells due to xenoma formation, their identity can only be hypothesised from our current knowledge. We believe that infection is primarily in the desmocytes, as infection is also seen along the haemocoelic divisions that have little or no such epidermal tissue layer. However, it is also possible that phagocytic haemocytes become infected, or are involved with the spread of the infection throughout the copepod, or are responsible for the initial transportation of spores from the portal of entry to the site of infection. Xenoma formation has not been reported for other members of the Enterocytozoonidae.

### Comparative ultrastructural features of Desmozoon and the Enterocytozoonidae (Table 2)

**Table 2:** Characteristics of the four microsporidian genera contained within the family Enterocytozoonidae

Feature	***Desmozoon *n. gen**.	*Enterocytozoon*	*Nucleospora*	*Enterospora*
Host	Caligid copepod, *Lepeophtheirus salmonis*	Mammals and birds^a ^and penaeid shrimp^b^	Fish (salmonid, freshwater, and marine)	Marine decapod crabs
Host cell-type infected	Desmocytes in the glycocalyx bordering haemocoelic cavities	Enterocytes ^a^ Tubule epithelial cells of the hepatopancreas ^b^	Haemopoietic cells and blood leukocytes ^c ^ Intestinal epithelial cells^d^	Hepatopancreas epithelial cells
Mature spore shape and size	Round to sub-spherical 2.34 × 1.83 (± 0.01) fresh	Spherical to ovoid, 1.5 × 0.8 ^a ^(fixed) Oval 1.1 × 0.7 (fresh/percoll-purified)	ovoid to pyriform, 2 × 1 (fixed) ^c ^ ellipsoidal 1.6 × 0.8 (ultrathin sections)^d^	1.3 (± 0.02) × 0.7 (± 0.01) (fixed)
Number of turns and type of polar filament	Isofilar, 5-8 turns (usually a double layer)	4-7 turns (double layer)^a ^ 5-6 turns (double layer)^b^	8-12 turns (single or double layer)^c ^ 4-5 turns (double layer) ^d^	4-5 turns (double layer)
Spore production	Polysporous, without interfacial envelop	Polysporous, without interfacial envelop	Polysporous, without interfacial envelop	Polysporous, without interfacial envelop
Location in host cell	Cytoplasm	Cytoplasm	Nucleoplasm (intranuclear)	Nucleoplasm (intranuclear)
Earliest stage observed	Diplokaryotic meront	Uninucleate meront	Uninucleate meront	Binucleate meront
Plasmodium/syncitium stage	Merogonial plasmodium divides via plasmotomy. No sporogonial plasmodium present.	Merogonial plasmodium transforms to sporogonial plasmodium without prior division.	Merogonial plasmodium transforms to sporogonial plasmodium without prior division.	Merogonial plasmodium transforms to sporogonial plasmodium without prior division.
Key references	Present study, [21,23]	^a ^[29,31,33,41] ^b ^[14]	^c ^[32,33] ^d ^[34]	[3,4]

^a ^*Enterocytozoon bieneusi*, ^b ^*Enterocytozoon hepatopenaei *

^c ^*Nucleospora salmonis*, ^d ^*Nucleospora secunda*

The diplokaryotic arrangement of nuclei seen in early merogony in *D. lepeophtherii *is not seen in the other genera from the Enterocytozoonidae, although it was reported in the initial description of *E. bieneusi *by Desportes et al., [29]. This early diplokaryotic nuclear formation in *E. bieneusi *was later rejected by Cali and Owen [41] who suggested it was an artifact caused by the flattening of the nuclei by adjacent ELIs. An early plasmodial stage of *Enterospora *sp. has been observed with 'paired nuclei' [3] but the nuclear arrangement does not appear to be typical of a microsporidian diplokaryon. The lack of complete descriptions of early merogonial stages for some members of the Enterocytozoonidae means that a brief or early diplokaryotic stage in the merogonial development cycle should not be completely ruled out for other members of the family.

All members of the Enterocytozoonidae share certain developmental features. Whilst early merogonial stages have not always been well characterized and remain somewhat contentious, all currently described species develop large sporogonial syncitia in which the polar filament extrusion apparatus forms and develops prior to eventual division to sporoblasts. This precocious development of the polar filament, prior to final division and its association with ELIs in large sporogonial plasmodia, are features that are unique to this family of microsporidia. In the three genera of the family: *Enterocytozoon*, *Nucleospora *and *Enterospora*, the sporogonial plasmodium develops directly from the merogonial plasmodium without division and continues to mature to form the characteristic sporogonial syncitium. This direct transformation is assumed to occur as no division of the multinucleated merogonial stages have been reported for these taxa, only their development and subsequent maturation to sporogonial forms.

Syncitial masses, such as merogonial and sporogonial plasmodia, are large proliferative stages that can occupy a significant proportion of the microsporidian-infected cell, which would make it unlikely for them to be missed during TEM examination of ultrathin sections of infected material. Therefore, it must be concluded that *D. lepeophtherii *develops without the formation of the sporogonial plasmodium that is typical for the family Enterocytozoonidae. Instead, the merogonial plasmodium divides to form uninucleate sporonts, which undergo a number of nuclear divisions, all of which are followed by cellular division, resulting in a final division to form two sporoblasts. The lack of the formation of the characteristic sporogonial plasmodium in *Desmozoon *might be considered sufficiently different for it not to be included in the Enterocytozoonidae. However, a precocious development of the polar filament can be found in young divisional sporonts (Fig 4a) and two sets of fully formed extrusion apparatus are sometimes found in large sporoblasts that are presumably yet to divide (Fig 4e). ELIs are also present throughout much of the developmental cycle in *Desmozoon*, being at their largest during late sporont and early sporoblast formation and reduced or absent in mature spores. Cali and Owen [41] suggested that the ELIs observed in *E. bieneusi *were storage vacuoles and probably involved in the formation of the polar filament and other spore structures. The ELIs in *Desmozoon *develop in close association with cytoplasmic cisternae and the developing polar filament (Fig 4c) supporting this hypothesis. They are also associated with the formation of the posterior vacuole, as they are positioned posteriorly in the developing spore ultimately where the posterior vacuole develops (Fig 4f). The presence of ELIs in *Desmozoon *during sporogony and the early development of the polar filament seen as early as young divisional sporonts are typical features of the Enterocytozoonidae that are seen in all the described species of the family and suggest that this microsporidian should be placed in the Enterocytozoonidae in spite of the lack of a sporogonial plasmodium.

Although some characteristics are present in all family members, there can be notable differences in the cellular processes involved in their formation. Morphogenesis of the polar filaments proceeds differently in the three described genera of the Enterocytozoonidae. In *E. bieneusi *a large electron dense body associated with vesiculotubular networks form electron dense disc-like structures (EDDs), which appear throughout the plasmodium and then fuse into arcs forming the polar filament coils [33,41]. In *Nucleospora *spp. the polar filament does not coalesce from EDDs but from cylinders or tubular structures that are associated with ribosome rich cytoplasm delimited by the ER cisternae [32,33]. In *Enterospora*, multiple spherical membrane-bound vesicles are present throughout the cytoplasm of the plasmodium [4], which presumably correspond to polar filament precursors and are similar to those seen in *E. bieneusi *although not as electron dense. The development of the polar filament in *D. lepeophtherii *is more similar to that of *Nucleospora *spp. as no typical EDDs develop and the polar filament develops within the cytoplasmic cisternae of ER (Fig 4a). However, the early assembly of the polar filament does not take place in the plasmodium as the merogonial plasmodium divides to form sporonts before being transformed to a sporogonial syncitium in this species.

Mature spores of *D. lepeophtherii *share many characteristics with those in the Enterocytozoonidae (Table 2). All members of the family have small round to ovoid (sub-spherical) spores, a single nucleus and between 4-8 turns of an isofilar polar filament, usually in a double coil, are polysporous and lack a complete interfacial envelope at all developmental stages.

### Molecular phylogenetics of the Enterocytozoonidae

*Desmozoon lepeophtherii *has been shown to be robustly supported as a member of the Enterocytozoonidae using phylogenetic analyses [[21], present study]. The Enterocytozoonidae form a small monophyletic group that is part of a larger clade described as being terrestrial in origin, but having evolved from ancestral freshwater microsporidians [42].

The Enterocytozoonidae has recently increased its size with respect to the number of genera and described species it contains. However, all the new descriptions are from aquatic marine crustacean hosts [3,4,14], including that in the present study. Unfortunately there are no DNA sequences available for the genus *Enterospora *infecting marine crabs, but ultrastructural descriptions adequately placed them in the Enterocytozoonidae. The increasing occurrence of microsporidia infecting crustacea in the Enterocytozoonidae is not unexpected, as a related gut-infecting microsporidian from *Daphnia *has been shown to be consistently placed as basal to the group [6,21]. Unfortunately no ultrastructural data is available for this parasite, but a similar microsporidian has been recently further documented from *Daphnia *populations in European lakes [7]. As more species are described and more gene sequences become available for the Enterocytozoonidae, some of these unresolved phylogenetic issues will hopefully be clarified. But, current phylogenetic analyses suggest that the Enterocytozoonidae is likely to have evolved from a common ancestor infecting a crustacean host. The phylogenetic importance of crustacean-infecting microsporidia in understanding the origins of species like *Enterocytozoon bieneusi *that infect humans may be significant.

### Other ultrastructural features of Desmozoon lepeophtherii in Lepeophtheirus salmonis

Numerous appendages/tubules were observed in the host cell cytoplasm during sporogony (Fig 4a-c). Takvorian and Cali [43] reviewed the form and function of microsporidian appendages, suggesting four different types. The appendages observed in this study most closely resemble the type I tubules described from *Glugea stephani *(Hagenmüller, 1899), but, during the present study, evidence that the tubules were continuous with the parasite plasmalemma was not apparent. The function of these appendages remains unknown but they have been suggested to facilitate host-parasite interchange of materials [43]. Microsporidian tubules have not been reported in the Enterocytozoonidae previously. They are rare amongst microsporidia that develop in direct contact with the host cell cytoplasm, but developing stages of both *E. bieneusi *and *E. hepatopenaei *have been observed with numerous tubule-like cytoplasmic projections extending into the host cell cytoplasm [14,33]. The lack of distinct cytoplasmic tubules in other members of the Enterocytozoonidae may be due to the common features of their sporogony resulting in the presence of sporogonial syncitia in all cases. The microsporidian tubules in *D. lepeophtherii *are only associated with sporogony and are more prominent during early sporogony, particularly in divisional young sporonts which are absent in other members of the family. Furthermore, microsporidian tubules have never been reported for intranuclear microsporidians. Tubule formation is not typically associated with xenoma-forming microsporidia and is more often observed in species that develop complete interfacial envelopes [44].

The microsporidian infection in *L. salmonis *does indeed complete its developmental cycle lacking any type of complete interfacial envelope. However, during late sporogony there were membranous secretions regularly observed near the outer surface of sporoblasts, occasionally in multiple layers (Fig 5a-b). These are thought to be parasite-derived secretions as no host cell remnants were associated with them. These membranous secretions were never seen to completely isolate the sporoblast from the host cell cytoplasm and hence cannot be considered an interfacial envelope. The structures were also observed as continuous concentric circular membranous whorls, whose function was not apparent (Fig 5c). These membranous secretions are possibly partially formed parasite derived interfacial envelopes or associated with or derived from the cytoplasmic tubules present during early sporogony. Similar non-persistent parasite derived interfacial envelopes have been reported from *Vairimorpha necatrix *(Kramer, 1965) where envelopes are formed during sporogony, which only sometimes develop to fully enclose the sporoblasts [44]. Similar membranous structures, described as type IV cytoplasmic tubules, have also been observed in sporophorous vesicles during sporogony in *V. necatrix *[44,45].

The intranuclear inclusions observed in infected host cells had an appearance consistent with either crystalline inclusions or viral like particles VLPs [46] (Fig 5d). Microsporidian infections have previously been associated with simultaneous viral infections. Both *Nucleospora *and *Enterocytozoon *have been reported to be associated with retroviral infection of their hosts. *Nucleospora salmonis *has been found in fish infected with the retrovirus that is believed to be the cause of plasmacytoid leukaemia [47,48], and *E. bieneusi *is found predominately in HIV-infected patients, although this is largely accepted as opportunistic infections due to a lowering of the immune status caused by the viral infection [49]. Intranuclear and intracytoplasmic crystalline inclusions are frequently associated with other cell abnormalities such as tumour cells and leukaemic conditions; intranuclear crystalline and paracrystalline inclusions have also been regularly associated with virally infected cells [46]. The VLPs seen in the present study are not thought to be related to the microsporidian infection but could warrant further study.

### Taxonomic summary

Phylum: Microsporidia (Balbiani, 1882)

Class: Microsporea (Levine & Corliss, 1963)

Order: Microsporida (Balbiani, 1882)

Family: Enterocytozoonidae (Cali & Owen, 1990)

### Specific diagnosis

Spores are round to sub-spherical, with an isofilar polar filament with 5-8 turns in a double layer. Entire development is in direct contact with the host cell cytoplasm. Large xenomas develop from the glycocalyx border that is immediately basal to the epidermal tissue layer beneath the cuticle, and desmocytes are thought to be the most likely host cell infected. Early diplokaryotic meronts develop into large plasmodia, with unpaired nuclei that divide by plasmotomy to form sporonts. During sporogony there is a precocious development of the polar filament extrusion apparatus which is associated with electron lucent inclusions. The polar filament can be fully arranged and well developed prior to the final division to form two sporoblasts. SSU rDNA sequence data is most similar to members of the *Nucleospora *and its position is robustly supported within the Enterocytozoonidae.

Type host: *Lepeophtheirus salmonis *(Krøyer) ex *Salmo salar *L.

Location: Numerous Atlantic salmon farms on the west coast of Scotland.

Type location: Toward Point, Argyll. Grid reference NS 137 672 GB (55° 51' N, 4° 58' W).

Site of infection: Desmocytes associated with the glycocalyx border, basal to the epidermal tissue layer under the cuticle and along haemocoelic divisions.

Etymology: The genus name *Desmozoon *refers to the cell type infected. Desomcyte: any elongated interstitial cell, as in fibrocyte or fibroblast. The specific name *lepeophtherii *refers to the generic name of the parasitic copepod host (*Lepeophtheirus*).

Type material: Two hapantotype ultrathin TEM sections have been submitted to the collections of the Natural History Museum, London, and assigned the accession numbers 2009: 11: 18:1 and 2009: 11: 18: 2

## Conclusion

The presence of a diplokaryotic stage during early merogony that undergoes nuclear dissociation and develops to form a merogonial plasmodium that divides to yield uninucleate sporonts has not been observed in the Enterocytozoonidae before. However, the typical family traits of the precocious development of the polar filament and the presence of ELIs were consistently observed, albeit in sporonts instead of a sporogonial plasmodium. Phylogenetic analyses consistently and robustly place *Desmozoon lepeophtherii *with other members of the Enterocytozoonidae as a monophyletic group, supporting the ultrastructural findings. This study highlights the importance of supplying a SSU rDNA sequence when describing new species of microsporidia. It is increasingly apparent that certain ultrastructural features, such as the diplokaryotic arrangement of nuclei can be lost or gained very rapidly over evolutionary time [42] and, following such characters leads to unacceptable polyphyletic groupings defined by character states that have evolved separately on numerous occasions and are not derived from a common ancestor [42]. Therefore the use of gene sequences to infer phylogenetic relatedness is extremely valuable and is vital for resolving the higher taxonomic issues in the microsporidia.

These results of this study lead us to the conclusion that the microsporidian hyperparasite of *L. salmonis *should be included in the Enterocytozoonidae. But, due to the unique ultrastructural features observed, xenoma formation, the novelty of the parasitic copepod host and the 10% sequence divergence seen in the SSU rDNA to its closest relative, that a new genus should be erected; the name *Desmozoon lepeophtherii *n. gen. n. sp. is proposed.

## List of abbreviations

TEM: transmission electron microscopy; SEM: scanning electron microscopy; SSU rDNA: small subunit ribosomal DNA; SSU: small subunit; ELIs: electron lucent inclusions; EDD: electron dense disc-like structures; ER: endoplasmic reticulum; VLPs: virus-like particles.

## Competing interests

The authors declare that they have no competing interests.

## Authors' contributions

MF collected material and prepared and performed the TEM and DNA analyses. MF and CS analysed the data and prepared the manuscript.
